# Oleoylethanolamide enhances regulatory T Cell function to accelerate plaque regression in atherosclerosis *via* PPARα activation

**DOI:** 10.1016/j.jbc.2026.113236

**Published:** 2026-06-04

**Authors:** Tong Ren, Yanchao Jiao, Le Zhang, Yijun Liu, Xilin Jiang, Lu Peng, Changsheng Yan, Jianbo Jia, Xin Jin

**Affiliations:** 1 Xiamen Cardiovascular Hospital of Xiamen University, School of Medicine, Xiamen University, Xiamen, China; 2 Department of Otolaryngology, Zhongshan Hospital of Xiamen University, School of Medicine, Xiamen University, Xiamen, China; 3 Department of Emergency, Huashan Hospital, Fudan University, Shanghai, China; 4 Department of Breast Surgery, Zhongshan Hospital of Xiamen University, School of Medicine, Xiamen University, Xiamen, China

**Keywords:** OEA, atherosclerosis, PPARα, RORγt

## Abstract

Regulatory T cells (Tregs) are essential for maintaining immune balance and limiting inflammatory damage within atherosclerotic plaques. Although the endogenous lipid mediator oleoylethanolamide (OEA) has reported anti-inflammatory and metabolic benefits, its effects on Treg differentiation and function during atherosclerosis are incompletely defined. Here, we tested OEA using *in vitro* naive CD4^+^ T-cell polarization assays and *in vivo* atherosclerosis models. OEA increased CD25^+^Foxp3^+^ Treg differentiation in polarization cultures and shifted the Treg compartment in atherosclerotic mice toward a more functional phenotype. PPARα dependence was supported by pharmacologic inhibition with MK886 and by genetic loss of PPARα, both of which abrogated OEA-induced Treg differentiation and functional enhancement. Mechanistically, OEA engaged a PPARα–RORγt pathway consistent with suppression of RORγt-associated programs during Treg differentiation. In therapeutic studies, adoptive transfer of OEA-conditioned Tregs promoted regression of established atherosclerotic plaques. Together, these data identify OEA as a modulator of Treg differentiation and activity and support its potential as a PPARα-dependent strategy to promote plaque regression and immune homeostasis in atherosclerosis.

Atherosclerosis is a progressive and chronic inflammatory disease characterized by the accumulation of lipids, proteins, calcifying materials, and inflammatory cells within the arterial walls, leading to the formation of plaques (1). These plaques can significantly narrow arteries, restrict blood flow, and result in severe cardiovascular events such as heart attacks and strokes (2, 3). Central to the pathogenesis of atherosclerosis is a dysregulated immune response that contributes to the chronic inflammation associated with this condition (1, 4). Among the diverse types of immune cells involved, regulatory T cells (Tregs) play a crucial role in maintaining immune homeostasis (5, 6, 7, 8). By suppressing excessive immune activation and promoting tolerance, Tregs are essential in mitigating the inflammatory processes that drive plaque development and progression (7, 8). Given their considerable atheroprotective potential, strategies that enhance Treg differentiation and function represent promising therapeutic avenues for atherosclerosis.

Oleoylethanolamide (OEA) is an endogenous lipid mediator, naturally occurring in animal and human tissues, and has garnered significant attention for its pleiotropic biological effects (9, 10). Previous research has primarily focused on its metabolic benefits, including the regulation of feeding behavior, lipid metabolism, and energy homeostasis, which are largely mediated through the activation of the nuclear receptor peroxisome proliferator-activated receptor alpha (PPARα) (10). Beyond its metabolic roles, OEA possesses potent anti-inflammatory properties. It has been demonstrated to inhibit the expression of adhesion molecules and pro-inflammatory cytokines in endothelial cells and to attenuate hepatic inflammation and oxidative stress (11, 12, 13, 14, 15). However, despite these established anti-inflammatory effects, its direct impact on the adaptive immune system, particularly on the differentiation and function of T lymphocyte subsets in the context of atherosclerosis, remains largely unexplored. The unique potential of OEA to enhance Treg function becomes more important in light of its selective activation of specific pathways, which could lead to therapeutic benefits without broad immunosuppression.

Building upon these insights, we hypothesized that OEA could modulate the immune response in atherosclerosis by specifically promoting the differentiation and enhancing the function of Treg cells. To test this, we examined the effects of OEA on T cell polarization *in vitro* and in disease-relevant models. We also investigated the molecular mechanism behind these effects using pharmacological and genetic approaches and evaluated whether OEA-treated Tregs could promote plaque regression. Our study reveals that OEA acts as a selective booster of Treg differentiation and function through a PPARα-dependent mechanism that stabilizes the expression of RORγt within Tregs. This work not only identifies a novel immunomodulatory function of OEA but also unveils a new pharmacological strategy for the treatment of atherosclerosis by harnessing the body's own regulatory mechanisms.

## Results

### OEA promotes Treg cell differentiation

**Figure 1:**
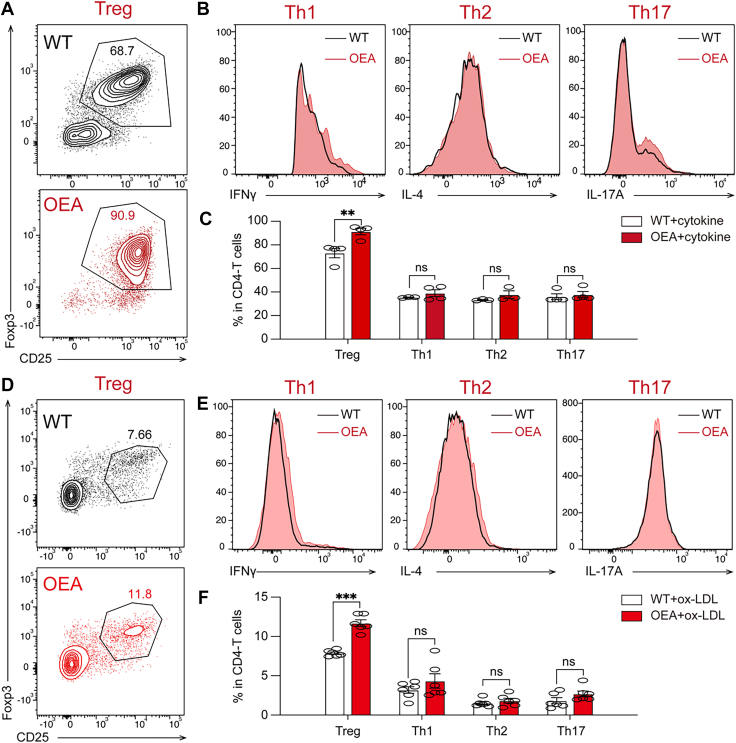
**OEA selectively promotes Treg differentiation *in vitro*.***A–C*, naive CD4+ T cells isolated from mouse lymph nodes were cultured under Treg-, Th1-, Th2-, or Th17-polarizing conditions in the absence or presence of OEA (20 μM) for 5 days (A) Representative flow cytometry plots and (*C*) quantification of CD4^+^CD25^+^Foxp3^+^ Tregs (n = 4 per group). *B*, representative flow cytometry plots and (*C*) quantification of Th1 (IFN-γ^+^), Th2 (IL-4^+^), and Th17 (IL-17A^+^) cells under their respective polarizing conditions with or without OEA (vehicle: n = 3 for Th1, n = 3 for Th2, n = 4 for Th17; OEA: n = 4 for Th1, n = 3 for Th2, n = 4 for Th17). *D–F*, splenocytes were stimulated with ox-LDL (100 μg/ml) in the presence of IL-2, with or without OEA (20 μM), for 48 h. *D*, representative flow cytometry plots and (*F*) quantification of induced Tregs (n = 6 per group). *E*, Representative flow cytometry plots and (*F*) quantification of other T helper subsets (n = 6 per group). Data are presented as mean ± SEM. ∗∗*p* < 0.01, ∗∗∗*p* < 0.001, ^ns^P > 0.05 by unpaired two-tailed Student's *t* test.

Naive CD4 T cells play a crucial role in the immune response, and their differentiation into various subtypes is significant in the development and progression of atherosclerosis. We investigated the effect of OEA on CD4^+^ T-cell behavior by activating naive CD4^+^ T cells isolated from lymph nodes *in vitro*. Our findings revealed that OEA significantly enhanced the conversion of naive T cells into Treg cells, achieving transformation rates substantially higher than those in the control group (Fig. 1, *A* and *C*), in a time- and dose-dependent manner (Fig. S1). In contrast, OEA did not significantly affect the differentiation of Th1, Th2, and Th17 cells (Fig. 1, *B* and *C*), underscoring OEA’s specific promoting effect on Treg cells.

To further assess the relevance of OEA in an atherosclerosis-like environment, we stimulated splenocytes with ox-LDL. Consistent with our initial results, OEA treatment in this context also led to increased Treg differentiation without altering the differentiation of other T helper cells (Fig. 1, *D* and *F*). These results demonstrate that OEA specifically promotes the differentiation of Treg cells, suggesting its potential to modulate immune responses in the context of atherosclerosis.

### OEA treatment promotes Treg differentiation and function

**Figure 2:**
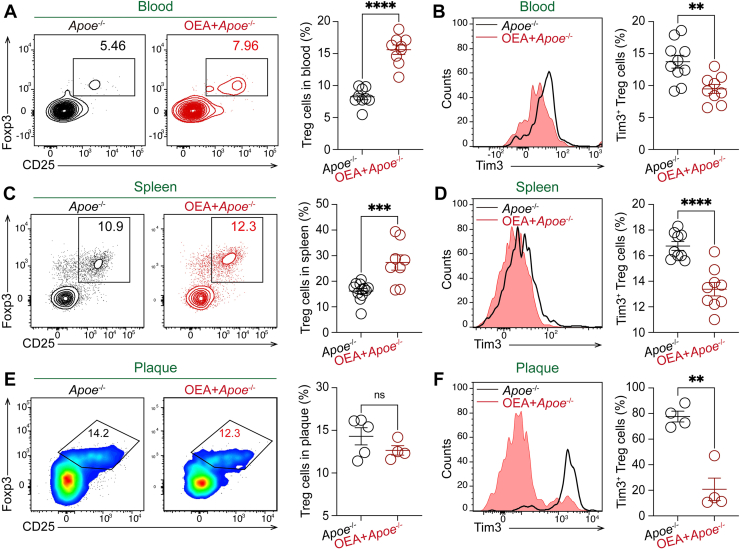
**OEA treatment expands functional Tregs and reduces dysfunctional Tregs in atherosclerotic mice.***Apoe*^*−/−*^ mice were fed a high-fat diet (HFD) for 20 weeks and treated with OEA (30 mg/kg, i.p., once daily) or vehicle. *A, C*, and *E*, frequency of CD4^+^CD25^+^Foxp3^+^ Tregs in (*A*) blood (n = 9 per group), (*C*) spleen (vehicle: n = 12; OEA: n = 8), and (*E*) atherosclerotic plaques (vehicle: n = 5; OEA: n = 4). *B, D*, and *F*, frequency of Tim3^+^ Tregs within the CD4^+^Foxp3^+^ population in (*B*) blood (vehicle: n = 10; OEA: n = 9), (*D*) spleen (n = 9 per group), and (*F*) atherosclerotic plaques (n = 4 per group). Data are presented as mean ± SEM. ∗∗*p* < 0.01, ∗∗∗*p* < 0.001, ∗∗∗∗*p* < 0.0001, ^ns^P > 0.05by unpaired two-tailed Student's *t* test.

The above results inspired us to further investigate the function of OEA in T cell–mediated atherosclerosis. Specifically, we examined the impact of OEA on Treg cell numbers and function in different tissues of atherosclerosis mice (*Apoe*^−/−^ mice fed HFD). We compared these with OEA-treated counterparts. First, our analysis of T, B, CD4^+^, and CD8^+^ cell populations in the spleen and blood showed no significant differences between OEA-treated and untreated mice, indicating that OEA does not alter the development of peripheral T lymphocytes (Fig. S2). However, the percentage of Treg cells was notably higher in the blood and spleen of OEA-treated mice (Fig. 2, *A* and *C*). Additionally, there was a significant decrease in the number of dysfunctional Tregs, identified by Tim3 expression, in these mice (Fig. 2, *B* and *D*). These findings suggest that OEA enhances both the differentiation and the functional quality of Treg cells in peripheral circulation in the context of atherosclerosis. Within the atherosclerotic plaque, although the overall proportion of Treg cells was comparable between treated and untreated groups, the presence of dysfunctional Tregs was significantly reduced in OEA-treated mice (Fig. 2, *E* and *F*). This reduction implies that OEA enhances the functional efficacy of Treg cells within the plaque, promoting their active role in exerting atheroprotective functions.

### OEA-mediated PPARα promotes Treg differentiation in atherosclerosis mice

**Figure 3:**
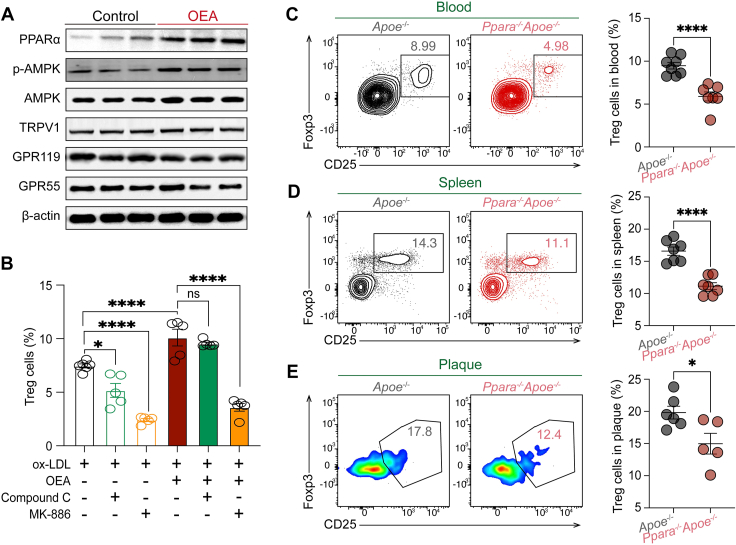
**OEA enhances Treg differentiation through a PPARα-dependent mechanism.***A*, immunoblot analysis of PPARα, phospho-AMPK (p-AMPK), total AMPK (t-AMPK), TRPV1, GPR119, and GPR55 in cells cultured under Treg-polarizing conditions with or without OEA (20 μM) for 5 days (n = 3 per group). *B*, flow cytometric analysis of induced Tregs from splenocytes stimulated with ox-LDL and IL-2 and treated with OEA (20 μM), MK886, Compound C, or the indicated combinations (ox-LDL: n = 6; all other groups: n = 5). *C–E*, *Ppara*^*−/−*^*Apoe*^*−/−*^ and *Apoe*^*−/−*^control mice were fed an HFD for 20 weeks. Frequency of CD4^+^Foxp3^+^ Tregs in (*C*) blood (*Apoe*^*−/−*^: n = 8; *Ppara*^*−/−*^*Apoe*^*−/−*^: n = 7), (*D*) spleen (n = 7 per group), and (*E*) atherosclerotic plaques (*Apoe*^*−/−*^: n = 6; *Ppara*^*−/−*^*Apoe*^*−/−*^: n = 5). Data are presented as mean ± SEM. ∗*p* < 0.05, ∗∗∗∗*p* < 0.0001, ^ns^P > 0.05 by one-way ANOVA with Tukey's *post hoc* test (*B*) or unpaired two-tailed Student's *t* test (*C–E*).

OEA acts through several targets, including PPARα, AMPK, TRPV1, GPR119, and GPR55. To uncover the molecular mechanisms by which OEA influences Treg cell differentiation, we analyzed the expression of proteins targeted by OEA in induced Treg cells. Our analysis revealed a significant increase in PPARα protein levels and AMPK phosphorylation in Treg cells differentiated from naive CD4^+^ T cells upon OEA stimulation (Fig. 3*A*). To investigate the roles of PPARα and AMPK in OEA-induced Treg differentiation within an atherosclerosis-like environment, we applied pharmacological inhibitors to splenocytes activated with ox-LDL. MK886, which disrupts the PPARα pathway, and compound C, which inhibits AMPK phosphorylation, were both used. We observed that Treg cell differentiation was downregulated in the presence of these inhibitors, even without OEA treatment (Fig. 3*B*). Interestingly, the inhibition of PPARα led to a significant reduction in induced Treg differentiation in both OEA-treated and untreated samples (Fig. 3*B*). In parallel, silencing AMPK also promoted Treg differentiation in OEA-treated cells (Fig. 3*B*). These findings suggest that OEA primarily targets PPARα to facilitate Treg cell differentiation.

To further assess the role of PPARα in regulating T cells and atherosclerosis *in vivo*, we generated *Ppara*^*−/−*^Apoe^−/−^double knockout mice. *Apoe*^*−/−*^ mice fed a high-fat diet and treated with OEA (30 mg/kg/day, i.p.) developed significantly smaller *en face* oil red O-positive lesions in the aorta compared with vehicle-treated controls, but this protective effect was abolished in *Ppara*^*−/−*^*Apoe*^*−/−*^ mice. These data strongly suggest that the atheroprotective effect of OEA is largely, if not completely, PPARα-dependent (Fig. S3). Following a HFD, *Ppara*^*−/−*^*Apoe*^*−/−*^ mice exhibited significantly lower percentages of Treg cells in the blood, spleen, and atherosclerotic plaques compared to controls (Fig. 3, *C*–*E*). Moreover, in naive CD4+ T cells lacking PPARα, OEA failed to enhance Treg differentiation *in vitro* (Fig. S4). Collectively, these findings indicate that OEA promotes Treg differentiation and atheroprotection predominantly through a PPARα-dependent mechanism.

### PPARα-mediated RORγt controls Treg differentiation

The differentiation pathways of Th17 and Treg cells exhibit significant plasticity, both originating from naive CD4^+^ T cell precursors *via* TGF-β signaling (16). The cytokine milieu critically determines their fate, with high concentrations of TGF-β promoting Foxp3 expression and Treg differentiation, while the presence of proinflammatory cytokines like IL-6 or IL-21 in combination with TGF-β promotes RORγt expression and Th17 differentiation (17). During early differentiation, some T cells may co-express Foxp3 and RORγt; these cells eventually stabilize into their respective lineages based on persistent environmental signals (18, 19). Interestingly, RORγt has been found to be essential for the stability and function of RORγt^+^ Treg cells, rather than disrupting the Treg lineage (19).

**Figure 4:**
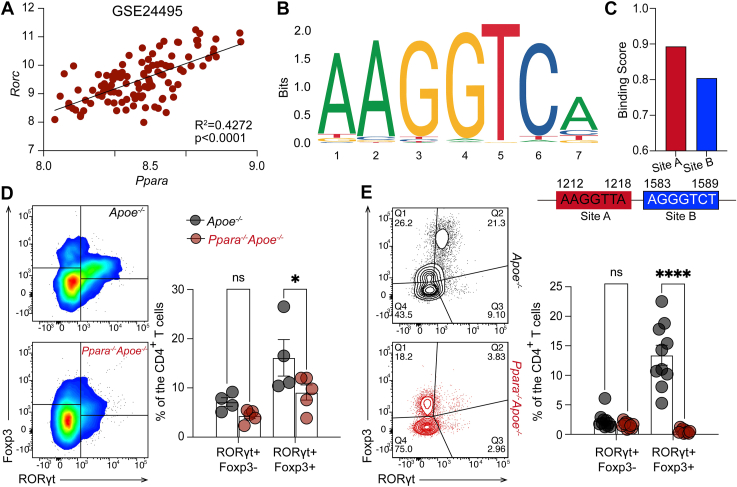
**PPARα is required for maintaining RORγt expression in Tregs within the atherosclerotic microenvironment.***A*, correlation analysis of *Ppara* and *RORC* expression in dataset GSE24495. *B*, *Ppara* transcription factor binding motif. *C*, predicted PPARα binding sites in the RORγt regulatory region (positions 1212–1218 and 1583–1589). *D* and *E*, frequency of RORγt positive cells in CD4^+^CD25^+^Foxp3^+^ and CD4^+^CD25^+^Foxp3^-^ T cells from (*D*) atherosclerotic plaques (*Apoe*^*−/−*^: n = 4; *Ppara*^*−/−*^*Apoe*^*−/−*^: n = 5) and (*E*) spleen (n = 10 per group) after 20 weeks of HFD. Data are presented as mean ± SEM. ∗*p* < 0.05, ∗∗∗∗*p* < 0.0001, ^ns^P > 0.05 by unpaired two-tailed Student's *t* test.

In order to underline the regulatory axis between RORγt and PPARα, we analysis the linear relation of these two genes in GSE24495 which including the gene expression profiling of 113 human atherosclerotic plaque. Surprisingly, RORγt and PPARα had strong linear relation by Cor analysis (Fig. 4*A*). Further, we found that PPARα have the binding capcity in the upstream 1212 to 1218 sites and 1583 to 1589 sites (Fig. 4, *B* and *C*).

To explore whether PPARα influences Treg stability through modulation of the RORγt profile in an atherosclerotic environment, we assessed RORγt levels in CD4^+^CD25^+^ T cells from the plaque and spleen of HFD-fed *Apoe*^−/−^ mice and *Ppara*^*−/−*^*Apoe*^−/−^ mice. Our findings show a marked reduction in RORγt levels within CD4^+^CD25^+^Foxp3^+^ T cells in both the plaque and the spleen of PPARα-deficient mice, while no significant change was observed in CD4^+^CD25^+^Foxp3^-^ T cells (Fig. 4, *D* and *E*). These observations suggest that RORγt is involved in Treg cell differentiation and that PPARα plays a crucial role in regulating RORγt expression within Treg cells, thereby influencing their stability and function in an atherosclerotic context.

### OEA-stimulated Treg cells promote the regression of atherosclerotic plaques

**Figure 5:**
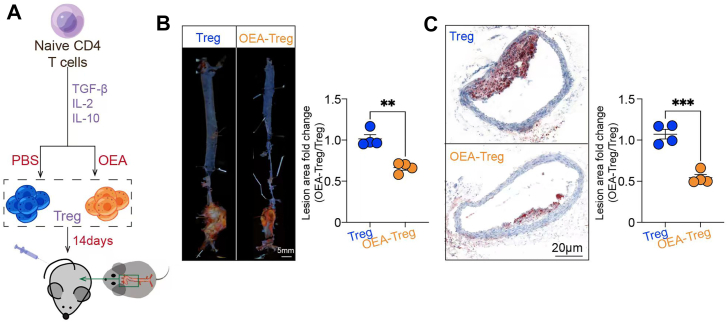
**Adoptive transfer of OEA-stimulated Tregs promotes regression of established atherosclerotic plaques.***A*, schematic of the aortic transplant regression model: aortic arches from atherosclerotic *Apoe*^*−/−*^donor mice were transplanted into normocholesterolemic C57BL/6J recipients, followed by intravenous transfer of *ex vivo* OEA-stimulated Tregs or vehicle-stimulated control Tregs. *B*, representative images and quantification of aortic Oil Red O staining (n = 4 per group). *C*, representative images and quantification of Oil Red O staining of aortic cross-sections (n = 4 per group). Scale bar = 5 mm. Data are presented as mean ± SEM. ∗∗*p* < 0.01, ∗∗∗*p* < 0.001 by unpaired two-tailed Student's *t* test.

**Figure 6:**
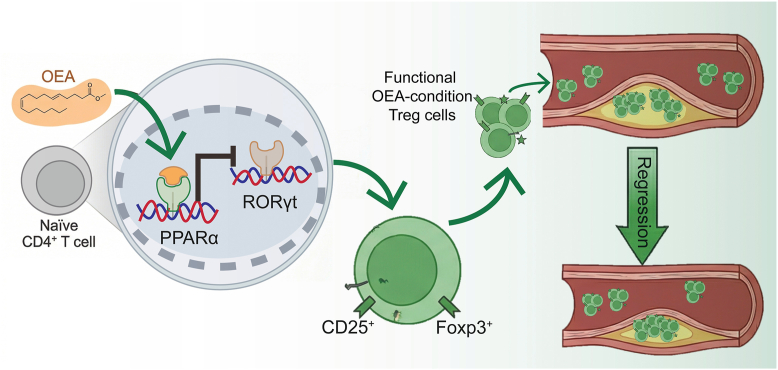
Mechanisms and Therapeutic Effects of OEA in Atherosclerosis: PPARα-Dependent Treg Enhancement.

Given the ability of Treg cells to suppress pro-inflammatory factors and ameliorate the inflammatory environment within atherosclerotic plaques, we investigated whether OEA-stimulated Treg cells could be linked to plaque regression. We employed an aortic transplant model of atherosclerosis regression, wherein the aortic arch from mice with advanced atherosclerosis (HFD-fed *Apoe*^−/−^ mice) was transplanted into normocholesterolemic C57BL/6 mice. Post-transplantation, these recipient mice were injected intravenously with either OEA-stimulated Tregs or non-stimulated Tregs (Fig. 5*A*). Our results revealed that mice treated with OEA-stimulated Tregs exhibited a significantly reduced lesion area in the aortic arch compared to those receiving non-stimulated Tregs (Fig. 5*B*). Furthermore, histological analyses corroborated these findings by demonstrating a marked reduction in plaque size in mice treated with OEA-stimulated Tregs (Fig. 5*C*). These outcomes indicate that OEA-stimulated Treg cells facilitate the regression of atherosclerotic plaques, highlighting their potential therapeutic utility in atherosclerosis regressing (Fig. 6).

## Discussion

Atherosclerosis is a chronic inflammatory disease driven by a dysregulated immune response, where Tregs play a crucial atheroprotective role by suppressing effector immune responses and maintaining immune tolerance (1, 8). While strategies to enhance Treg differentiation represent promising therapeutic avenues, achieving lineage-specific modulation without triggering broad immunosuppression remains a significant challenge. OEA has long been recognized as a protective endogenous lipid mediator in vascular and metabolic contexts. OEA has shown efficacy in reducing inflammation in THP-1 cells and limiting ox-LDL-induced necrosis in HUVECs (20, 21). Beyond the vascular wall, OEA demonstrates hepatoprotective effects in non-alcoholic fatty liver disease (NAFLD) models and broad metabolic benefits, such as ameliorating diet-induced obesity and modulating the gut microbiome (13, 14, 15, 22) However, its direct immunomodulatory effects on specific lymphocyte subsets in atherosclerosis have remained unexplored. A critical pharmacological consideration with OEA is its rapid hydrolysis into oleic acid by the enzyme FAAH. While oleic acid is an abundant free fatty acid known to broadly modulate immune cell metabolism, our data demonstrate that oleic acid completely fails to replicate the specific, robust Treg differentiation induced by OEA. This finding is paramount, as it establishes that the immunomodulatory effects are driven by intact OEA itself, rather than its downstream metabolite, underscoring OEA’s unique role as a highly specific immunometabolic signal. To rigorously define this pharmacological effect, we utilized a sensitive *in vitro* detection window by titrating TGF-β to a suboptimal concentration. This approach allowed us to clearly demonstrate that OEA drives Treg differentiation in a distinct time- and dose-dependent manner.

A key contribution of our study is the definitive elucidation of the PPARα-dependent mechanism underlying OEA’s effects. PPARα is widely recognized as a promiscuous lipid sensor that binds various endogenous free fatty acids, which typically exert modest and non-specific effects on T cells. Our study advances the current understanding of PPARα pharmacology by demonstrating that OEA acts as a high-affinity endogenous ligand capable of directing highly specific Treg enhancement. To provide definitive evidence that OEA’s biological effects are strictly dependent on PPARα and not mediated through off-target lipid receptors, we performed genetic loss-of-function experiments. Systemic administration of OEA significantly attenuated high-fat diet-induced plaque formation and expanded functional Tregs in native *Apoe*^*−/−*^ mice, but these protective effects were completely abolished in *Apoe*^*−/−*^*Ppara*^*−/−*^ mice. The ability of OEA to drive Treg differentiation was similarly lost in PPARα-deficient T cell cultures. These genetic exclusivity data provide the rigorous proof required to establish the OEA-PPARα axis as a specific and non-redundant pathway in Treg biology.

In addition to confirming target specificity, our findings uncover a novel mechanistic link between PPARα activation and the transcriptional upregulation of *Rorc* (encoding RORγt) within the Treg compartment. Accumulating literature highlights that Tregs residing in inflammatory microenvironments require additional transcriptional reinforcement to maintain their stability. RORγt + Tregs have recently been identified as a highly stable and suppressive subset capable of thriving in inflammatory tissues. In our aortic transplant regression model, we demonstrated that Tregs conditioned *ex vivo* with OEA directly upregulated the PPARα-RORγt axis. This OEA-driven transcriptional reprogramming is the crucial mechanism that equips the transferred Tregs with the enhanced stability required to survive and function within the harsh atherosclerotic milieu. Consequently, this establishes a direct causal relationship between OEA-stimulated Tregs and plaque regression.

From a translational perspective, our findings present a highly practical therapeutic strategy. Systemic delivery of OEA successfully expanded functional Treg populations, yet the clinical translation of OEA has historically been hindered by its relatively low oral bioavailability. Our aortic transplant regression model offers an elegant solution to this pharmacological limitation. By demonstrating that Tregs pre-treated with OEA are sufficient to directly accelerate the regression of established plaques, we propose a shift toward targeted cellular immunotherapy over systemic drug administration. Expanding patient-derived Tregs with OEA prior to reinfusion would bypass bioavailability constraints while directly leveraging OEA’s specific ability to enhance Treg stability *via* the PPARα-RORγt pathway. This approach could potentially yield a superior cellular product compared to current standard expansion protocols.

In conclusion, our study unveils a novel immunometabolic pathway that regulates T cell-mediated immunity in atherosclerosis. By distinguishing OEA from its FAAH metabolite and general free fatty acids, and by proving strict physiological dependency on PPARα using double-knockout mouse models, we provide a definitive mechanistic framework. We identify OEA as a specific physiological enhancer of Treg differentiation and function through PPARα-dependent stabilization of RORγt expression. Targeting the OEA-PPARα axis through direct pharmacological intervention, or utilizing OEA as an adjunct for Treg-based cellular therapy, holds significant promise for the treatment of atherosclerosis and other inflammatory diseases characterized by Treg dysfunction.

## Experimental procedures

### Mice

Male C57BL/6J mice and PPARα knockout mice (B6;129S4-*Ppara*^tm1Gonz^/J; The Jackson Laboratory, Bar Harbor, ME) were used. The PPARα knockout line had been backcrossed onto the C57BL/6J background for >10 generations and was therefore maintained on a C57BL/6J congenic background. Male apolipoprotein E–deficient mice (*Apoe*^−/−^) were obtained from Vital River Laboratory Animal Technology Co., Ltd (Beijing, China) (C57BL/6J background). *Ppara*^*−/−*^*Apoe*^*−/−*^ double-knockout mice were generated by crossing *Apoe*^*−/−*^ mice with *Ppara*^*−/−*^ mice and intercrossing the F1 offspring to obtain experimental cohorts; genotypes were confirmed by PCR. Unless otherwise indicated, age-matched male *Apoe*^*−/−*^ littermates (*Ppara*^*+/+*^*Apoe*^*−/−*^) were used as controls for the double-knockout experiments. Mice were acclimated for 1 week before experiments and were housed under specific pathogen–free conditions with ad libitum access to food and water under a 12-h light/dark cycle. Xiamen University's Institutional Animal Care and Use Committee approved the animal procedures.

### Isolation of naive T cells and *in vitr*o T Cell differentiation

Lymph nodes were harvested from euthanized male C57BL/6J mice (8 weeks old; CO_2_ asphyxiation) and processed into single-cell suspensions using standard methods. Naive CD4^+^ T cells were purified by fluorescence-activated cell sorting (FACS) as CD4^+^CD8^-^CD62L^hi^CD44^lo^ cells. Sorted cells were cultured in 96-well plates in lymphocyte medium (BasalMedia, T110 KJ) supplemented with 10% fetal bovine serum, 100 U/ml penicillin, 100 μg/ml streptomycin, 50 μM β-mercaptoethanol (Sigma-Aldrich, M3148), and CD3/CD28 T-Activator (Gibco, 11456D).

For polarization, cells were cultured under the following conditions: TGF-β1 (5 ng/ml, PeproTech) and IL-2 (5 ng/ml, Sigma-Aldrich) for Treg; IL-12 (10 ng/ml, R&D systems) and anti-IL-4 (10 μg/ml, clone 11B11, BioGems) for Th1; IL-4 (20 ng/ml, PeproTech) and anti-IFN-γ (10 μg/ml, clone XMG1.2, PeproTech) for Th2 (23); TGF-β1 (2 ng/ml, PeproTech), IL-6 (30 ng/ml, abs04084, absin, CN) for Th17 polarization (24). OEA (Sigma-Aldrich) was added at 20 μM. To maintain effective drug concentration during the 5-day culture, half of the culture medium was replaced with fresh medium containing OEA every 2 days. Differentiation efficiency was assessed on days 3 and 5 by flow cytometry as CD4^+^CD25^+^Foxp3^+^(Treg), CD4^+^CD44^+^IFN-γ^+^(Th1), CD4^+^CD44^+^IL-4^+^ (Th2), CD4^+^CD44^+^IL-17A^+^(Th17).

### Splenocyte isolation and stimulation

Splenocytes were isolated from euthanized male C57BL/6J mice (8 weeks old; CO_2_ asphyxiation), following established protocols (25). For activation, splenocytes were cultured with ox-LDL (100 ng/ml, Sigma-Aldrich) and IL-2 (5 ng/ml, Sigma-Aldrich) in the presence or absence of OEA (20 μM, Sigma-Aldrich) for 24 or 48 h. For the 48-h stimulation, half of the medium was replenished with fresh medium containing OEA at 24 h. PPARα antagonist MK886 (1 μM, Sigma-Aldrich) and AMPK inhibitor Compound C (10 μM, Sigma-Aldrich) were added 1 hour prior to OEA (26, 27).

### Atherosclerosis progression models

For the atherosclerosis progression model, male *Apoe*^*−/−*^ and *Ppara*^*−/−*^*Apoe*^*−/−*^ mice were started on a high-fat diet (HFD; 0.5% cholesterol, 42% kcal from fat) at 4 weeks of age and maintained on the diet for 20 weeks. Mice received OEA (30 mg/kg) or vehicle by intraperitoneal injection once daily throughout the 20-week feeding period. This dose was selected based on previous studies demonstrating efficacy without toxicity, and no adverse effects were observed in the treated mice throughout the experimental period. At the study endpoint, mice were euthanized by CO_2_ asphyxiation prior to tissue collection.

### Cell preparation and flow cytometry analysis

Single-cell suspensions were prepared from spleen, lymph nodes, blood and atherosclerotic plaques. Plaques were digested in RPMI-1640 containing 1 mg/ml Collagenase Type IV (Sigma) and 100 U/ml DNase I using a GentleMACS dissociator (Miltenyi Biotech). Leukocytes were enriched using a 42%/70% Percoll (Cytiva) density gradient centrifugation.

Cells were stained with fixable viability dye and fluorochrome-conjugated antibodies against surface markers (CD4, CD8, CD25, Tim-3). For intracellular staining (Foxp3, RORγt, IFN-γ, IL-4, IL-17A), cells were fixed and permeabilized using the Foxp3/Transcription Factor Staining Buffer Set (eBioscience) or Cytofix/Cytoperm kit (BD Biosciences). For cytokine detection, cells were re-stimulated with PMA (50 ng/ml) and ionomycin (1 μg/ml) in the presence of GolgiStop for 4 h prior to staining. Data were acquired on an LSRFortessa X-20 flow cytometer (BD Biosciences) and analyzed using FlowJo software (v10.8, Tree Star).

### Western blotting

Cells lysed in RIPA buffer with inhibitors had protein concentrations measured by a BCA assay. Proteins were separated by SDS-PAGE, transferred to PVDF membranes, blocked, and incubated with primary antibodies against PPARα, phospho-AMPKα (Thr172), total AMPKα, TRPV1, GPR119, GPR55, and β-Actin. Blots were visualized using an ECL substrate, with band intensities quantified using ImageJ software.

### Bioinformatic analysis of *Rorc* and *Ppara* in human atherosclerotic plaques

To explore the regulatory relationship between RORγt and PPARα in human atherosclerosis, we performed a bioinformatic analysis using a publicly available gene expression dataset. The gene expression profiles of 113 human atherosclerotic plaques were obtained from the Gene Expression Omnibus (GEO) dataset GSE24495. Linear correlation analysis between RORC (encoding RORγt) and PPARα gene expression levels was performed using Pearson correlation. To further investigate the potential direct regulatory mechanism, we analyzed the promoter region of the RORC gene using the JASPAR database (http://jaspar.genereg.net/).

### Treg cells transfer for plaque regression

Plaque regression experiments were performed essentially as described by Fisher and colleagues (28). Briefly, male *Apoe*^*−/−*^ mice were fed an HFD for 20 weeks, after which a subset of mice was euthanized to provide baseline lesions. Donor aortic arches from HFD-fed *Apoe*^*−/−*^ mice were transplanted into the infrarenal abdominal aorta of recipient male C57BL/6J mice (12 weeks old) under isoflurane anesthesia (1–2%). One week after transplantation, recipient mice received either OEA-stimulated or unstimulated Tregs (1 × 10^7^ cells per injection) by tail-vein injection every 2 days for 2 weeks.

### Histological analysis

Atherosclerotic lesions were assessed following standard procedures. The entire aorta was perfused with PBS, dissected, opened longitudinally, and stained with Oil Red O solution for 30 min. Photographs were taken using a digital camera, and the total aortic and lesional areas were measured with ImageJ software. For the aortic sinus lesions, the donor aorta was embedded in paraffin, sectioned through the aortic root, and stained with Oil Red O solution for 30 min. The areas of atheromatous lesions and the total aortic root area were quantified using Image-Pro Plus software (version 4.0, Media Cybernetics, Silver Spring, USA). At least three random sections were analyzed from each mouse.

### Statistical analysis

All data are presented as mean ± standard error of the mean (SEM). Statistical analyses were performed using GraphPad Prism 10.0 software. Comparisons between two groups were analyzed using an unpaired two-tailed Student's *t* test. Comparisons among multiple groups were analyzed by unpaired two-tailed Student's *t* test, one-way or two-way ANOVA followed by Bonferroni's or Tukey's *post hoc* test. A value of *p* < 0.05 was considered statistically significant (^ns^P > 0.05; ∗*p* < 0.05; ∗∗*p* < 0.01; ∗∗∗*p* < 0.001; ∗∗∗∗*p* < 0.0001).

## Data availability

Data available on request from the author.

## Supporting information

This article contains supporting information.

## Conflict of interest

The authors declare that they have no conflicts of interest with the contents of this article.
