# Supplementary material for: Oleoylethanolamide enhances regulatory T Cell function to accelerate plaque regression in atherosclerosis via PPARα activation
Source: J Biol Chem. 2026 Jun 4;302(7):113236. doi: 10.1016/j.jbc.2026.113236 (PMC13332470; doi:10.1016/j.jbc.2026.113236)
Supplement: Supplementary file [file mmc1.docx]

**Supplemental Table**

Table S1

| Antibodys | Source | Identifier |
| --- | --- | --- |
| Anti-PPARα (3B6/PPAR) | Invitrogen | Cat# MA1-822; RRID: AB_2165745 |
| Anti-Phospho-AMPK alpha (Thr172) (40H9) | CST | Cat# 2535; RRID: AB_331250 |
| Anti-AMPK alpha (23A3) | CST | Cat# 2603; RRID: AB_ 490795 |
| Anti-TRPV1 (BS397) | Abcam | Cat# ab203103; RRID: AB_2934112 |
| Anti-GPR119 | Abcam | Cat# ab75312; RRID: AB_10660856 |
| Anti-GPR55 | Abcam | Cat# ab203663; RRID: AB_ 10662620 |
| β-actin | Abcam | Cat# ab6276; RRID: AB_2223210 |
| APC anti-CD25 (PC61.5) | eBioscience | Cat#17-0251-82; RRID: AB_469366 |
| PE-cy7 anti-CD4 (GK1.5) | eBioscience | Cat#25-0041-82; RRID: AB_469576 |
| Percp-cy5.5 anti-CD8a (53-6.7) | eBioscience | Cat# 45-0081-82; RRID: AB_1107004 |
| PE anti-Foxp3 (FJK-16s) | eBioscience | Cat# 12-5773-82; RRID: AB_465936 |
| PE anti-IFN-gamma (XMG1.2) | eBioscience | Cat# 12-7311-82; RRID: AB_466193 |
| APC anti-IL17a (eBio17B7) | eBioscience | Cat# 17-7177-81; RRID: AB_763580 |
| PE-cy7 anti-IL4 (11B11) | eBioscience | Cat#25-7041-82; RRID: AB_2573520 |
| PE-cy7 anti-CD44 (IM7) | eBioscience | Cat# 25-0441-82; RRID: AB_469623 |
| eFluor™450 anti-CD366(TIM3) (8B.2C12) | eBioscience | Cat# 48-5871-82; RRID: AB_2574081 |
| BV421 anti-RORγt (Q31-378) | BD | Cat#562894; RRID: AB_2687545 |


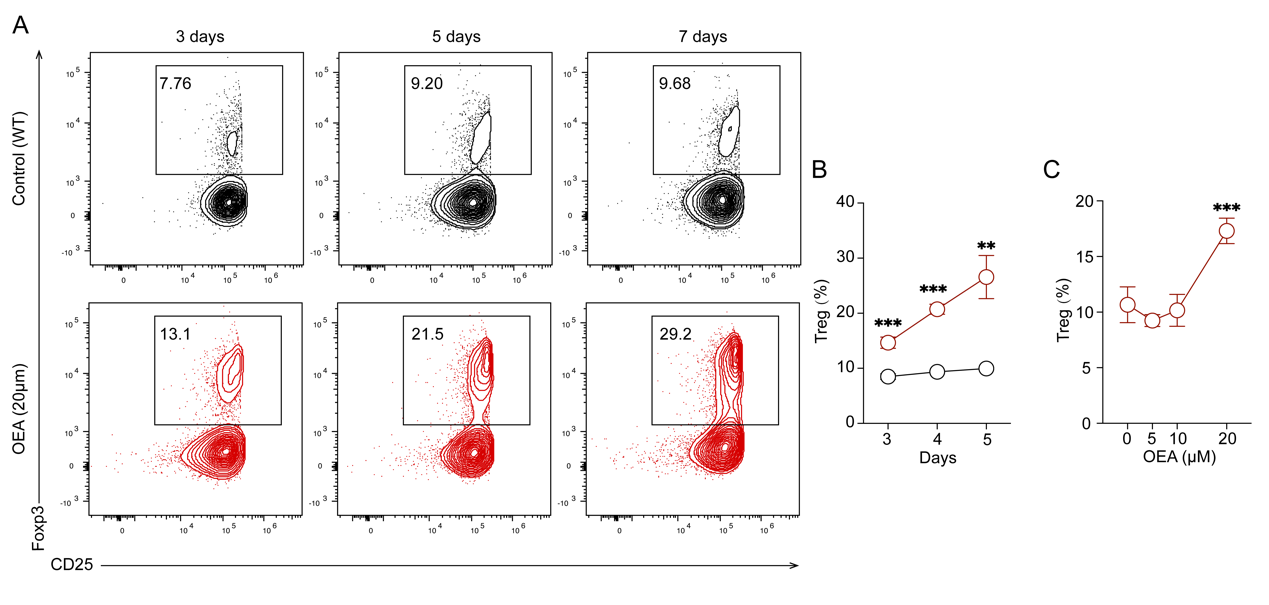


**Figure S1. OEA selectively promotes Treg differentiation in vitro**

(A–B) Naïve CD4+ T cells isolated from mouse lymph nodes were cultured under Treg-polarizing conditions in the absence or presence of OEA (20 μM) for 3-5 days. (A) Representative flow cytometry plots and (B) quantification of CD4^+^CD25^+^Foxp3^+^ Tregs for 3-5 days (n = 4 per group). (C) Quantification of Treg cells under with different concentration of OEA (0, 5, 10 and 20μM), n = 4 per group. Data are presented as mean ± SEM. ^**^P < 0.01, ^***^P < 0.001 by unpaired two-tailed Student's t-test.


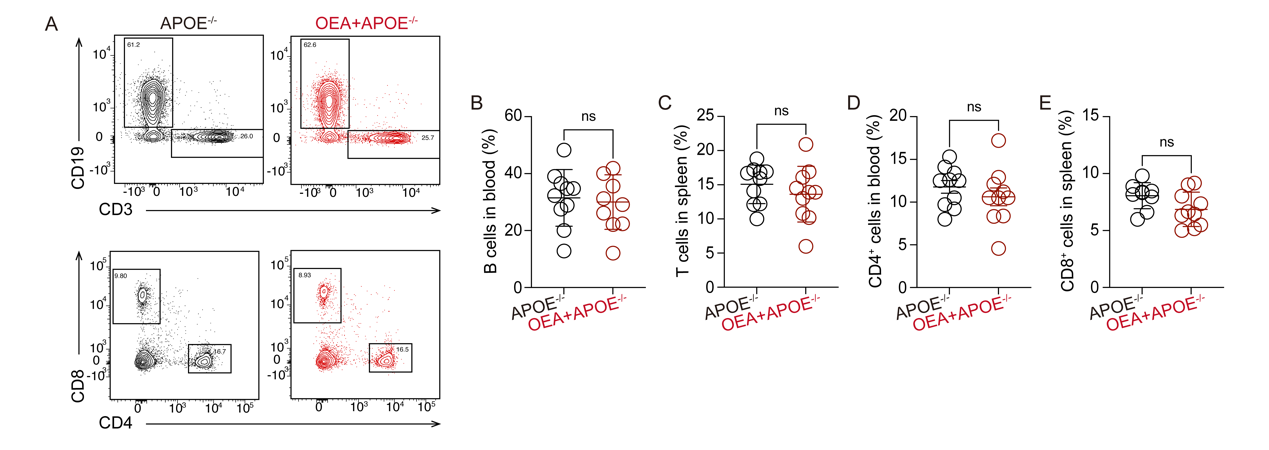


**Figure S2. OEA treatment does not alter the development of peripheral T lymphocytes**

ApoE^-/-^ mice were maintained on a high-fat diet (HFD) for 20 weeks and received daily intraperitoneal injections of 30 mg/kg OEA or vehicle (n = 9 for OEA; n = 10 for vehicle). (A-E) Flow cytometric analysis of the frequency of B cells (B), T cells (C), CD4-T cells (D) and CD8-T cells (E) in the spleen. Data are presented as mean ± SD. ^s^P>0.05 by unpaired two-tailed Student's t-test.


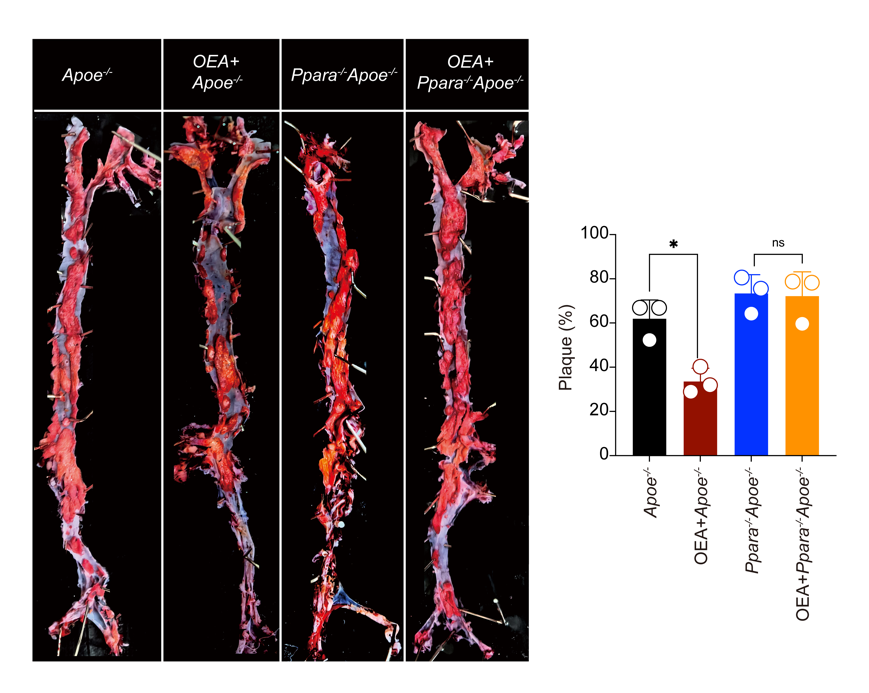


**Figure S3. The atheroprotective effect** of **OEA through a PPARα-dependent mechanism.**

*Apoe^-/-^ and Apoe^−/−^Ppara^−/−^* mice were fed a high-fat diet (HFD) for 20 weeks and treated with OEA (30 mg/kg, i.p., once daily) or vehicle. Representative images and quantification of aortic Oil Red O staining (n = 4 per group). Data are presented as mean ± SEM. ^*^P < 0.05, ^ns^P>0.05 by unpaired two-tailed Student's t-test.


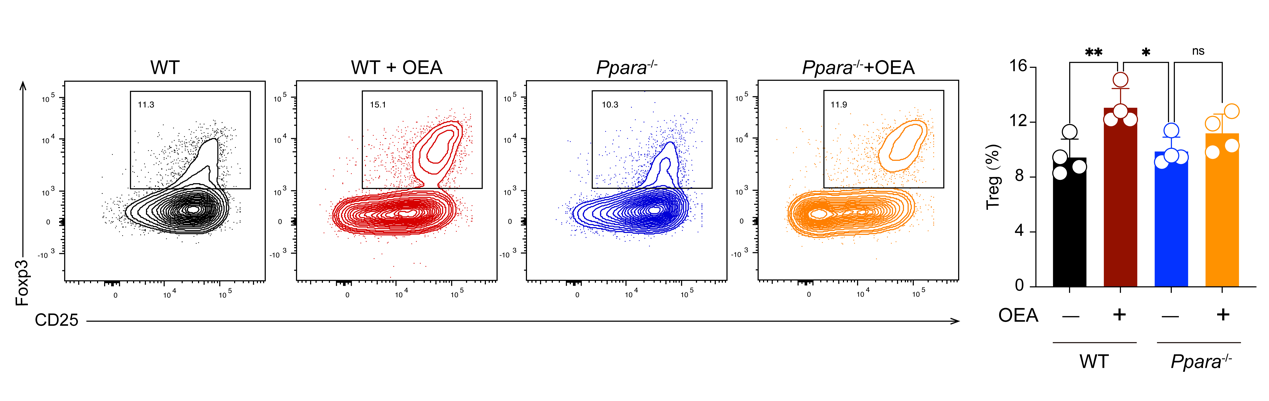


**Figure S4. OEA enhances Treg differentiation through a PPARα-dependent mechanism.**

Naïve CD4+ T cells isolated from WT and *Ppara*^-/-^ lymph nodes were cultured under Treg-polarizing conditions in the absence or presence of OEA (20 μM) for 3 days. Representative flow cytometry plots and quantification of CD4^+^CD25^+^Foxp3^+^ Tregs (n = 4 per group). Data are presented as mean ± SEM. ^*^P < 0.05, ^**^P < 0.01, ^ns^P>0.05 by one-way ANOVA with Tukey's post-hoc test (B) or unpaired two-tailed Student's t-test (C-E).


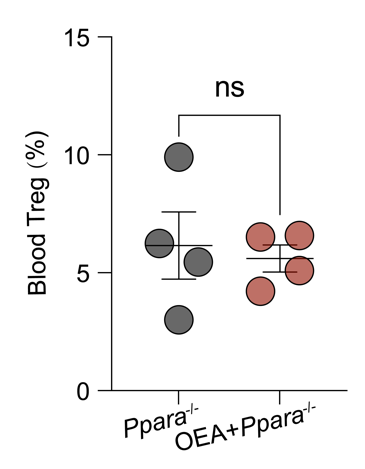


**Figure S4. OEA enhances Treg differentiation through a PPARα-dependent mechanism.**

*Ppara^-/-^Apoe^-/-^* mice were fed an HFD for 20 weeks and treated with OEA (30 mg/kg, i.p., once daily) or vehicle. Frequency of CD4^+^Foxp3^+^ Tregs in blood (n = 4 per group). Data are presented as mean ± SEM. ^ns^P>0.05by unpaired two-tailed Student's t-test.
